# Mesenchymal Stem Cells and their Derived Exosomes Promote Malignant Phenotype of Polyploid Non-Small-Cell Lung Cancer Cells through AMPK Signaling Pathway

**DOI:** 10.1155/2022/8708202

**Published:** 2022-04-04

**Authors:** Lili Wang, Mingyue Ouyang, Sining Xing, Song Zhao, Shuo Liu, Lingyan Sun, Huiying Yu

**Affiliations:** Laboratory of Basic Medicine, General Hospital of Northern Theatre Command, Shenyang, Liaoning 110016, China

## Abstract

Chemotherapy is an important method for the treatment of non-small-cell lung cancer (NSCLC), but it can lead to side effects and polyploid cancer cells. The polyploid cancer cells can live and generate daughter cancer cells via budding. Mesenchymal stem cells (MSCs) are pluripotent stem cells with repair and regeneration functions and can resist tissue damage caused by tumor therapy. This study is aimed at investigating the effects of MSCs and their derived exosomes on the biological characteristics of polyploid NSCLC cells and the potential mechanisms. We found that MSC conditioned medium (CM), MSCs, and MSC-exosomes had no effect on cell proliferation of the polyploid A549 and H1299 cells. Compared with the control group, MSCs and MSC-exosomes significantly promoted epithelial mesenchymal transformation, cell migration, antiapoptosis, and autophagy in the polyploid A549 and H1299 by activating AMPK signaling pathway, but no significant changes were observed in MSC-CM treatment. These results revealed that MSCs and MSC-exosomes promoted malignant phenotype of polyploid NSCLC cells through the AMPK signaling pathway.

## 1. Introduction

Lung cancer is one of the malignant tumors with the highest morbidity and mortality in the world, among which non-small-cell lung cancer (NSCLC) accounts for about 85% of the total lung cancer [1]. Chemotherapy and radiotherapy are commonly used in the treatment of lung cancer, but the consequent side effects such as bone marrow suppression, gastrointestinal mucosal injury, lung, and skin injury are common [2–5]. Recent research evidence shows that antitumor treatments such as chemotherapy can significantly increase the proportion of polyploid cancer cells in vivo [6, 7]. The polyploid cancer cells are resistant to chemotherapy and can produce progeny with active proliferation capabilities in vitro, thereby leading to tumor recurrence [8, 9].

Mesenchymal stem cells (MSCs) are pluripotent stem cells with repair and regeneration functions, which may be a new strategy for the treatment of side effects after radiotherapy and chemotherapy. Autologous MSCs, combined with hematopoietic stem cell transplantation, can promote hematopoietic system reconstitution after radiotherapy for breast cancer [10]. MSCs can home into the damaged mucosa, inhibit apoptosis of the small intestinal stem cells, and promote their proliferation and rapid repair of the damaged mucosa [11]. In addition, intravenous infusion of adipose-derived MSCs can alleviate acute radiation lung injury in rats through anti-inflammatory, antifibrotic, and antiapoptotic mechanisms [12]. Although MSCs can resist tissue damage caused by tumor therapy, whether they can simultaneously cause tumor recurrence is uncertain.

Exosomes are novel ways that accelerate communication among cells. In recent years, the interaction between MSCs derived exosomes and tumor cells has become one of the focus points in the field of tumor research. Recent research evidences have showed that MSC-derived exosomes promoted the proliferation and migration of breast cancer cells in vitro [13, 14]. However, Lang et al. concluded that miR-124a transfection of MSC-derived exosomes can significantly reduce the survival rate of glioblastoma cells and improve the survival rate of glioblastoma in mice [15]. Therefore, it is necessary to study the induction effect of MSCs and their derived exosomes on the residual polyploid NSCLC cells, subsequent to radiotherapy and chemotherapy.

In this study, we proposed to establish a model of polyploid NSCLC cells in vitro by inducing NSCLC cell lines A549 and H1299 with docetaxel (Doc), a traditional chemotherapeutic agent. The biological characteristics involved in proliferation, migration, apoptosis, and autophagy of polyploid NSCLC cells, induced by MSCs and their derived exosomes, were analyzed and the possible mechanism(s) discussed. This study is aimed at providing a theoretical basis for the safety of MSCs and their derived exosomes using in NSCLC patients with tissue and organ damage caused by radiotherapy and chemotherapy.

## 2. Material and Methods

### 2.1. Reagents and Antibodies

All the reagents and antibodies were purchased from different biotechnology companies. DMEM/F12 culture medium was from Basal Media biotechnology Inc. (Shanghai, China). Fetal Bovine Serum (FBS) was from Biological Industries Israel Beit Haemek Ltd. (Kibbuts Beit Haemek, Israel); Doc was from Selleckchem Inc. (Houston, TX, USA); Hochest 33342 was from Beyotime Institute of Biotechnology (Shanghai, China); propidium iodide (PI) was from Sigma-Aldrich (St. Louis, MO, USA); carboxyfluorescein diacetate succinimidyl ester (CFSE) was from BD Biosciences (San Jose, CA, USA); Annexin V-FITC/PI staining cell apoptosis detection kit was from Jiangsu Kaiji Biotechnology Co., Ltd; HLA-DR-FITC, CD14-APC, CD19-FITC, CD34-APC, CD45-PerCP, CD73-PE, CD90-PE, and CD105-PE were from BD biosciences (San Jose, CA, USA); the primary antibodies against human glyceraldehyde-3-phosphate dehydrogenase (GAPDH), matrix metalloproteinase 9 (MMP-9), B cell lymphoma-extra-large (Bcl-xl), Bcl-2 antagonist killer 1 (Bak), microtubule-associated protein 1 light chain 3 A/B (LC3A/B), Sequestosome 1 (P62), 5′-AMP-activated protein kinase (AMPK), and phospho-AMPK (Thr172) were from Cell Signaling Technology Inc; the second antibody against rabbit IgG (H + L) (horseradish peroxidase conjugate) and F(ab′)2 Fragment (Alexa Fluor® 488 Conjugate) were from Cell Signaling Technology Inc; the supersignal west pico chemiluminescent substrate was from Pierce Biotechnology Inc. (Rockford, Illinois, USA).

### 2.2. Cell Culture and Treatment Schedule

Human NSCLC cell lines A549 and H1299 were purchased from American Type Culture Collection (ATCC). The cells were resuscitated and cultured in DMEM/F12 culture medium supplemented with 10% FBS in a 37°C, 5% CO_2_ incubator. The cells from the 3^rd^ to the 4^th^ passage were taken for subsequent experiments. When the cell density reached about 60%-70%, cells were treated with Doc (0.5/1 *μ*M) for 24 hours and then allowed to recover for 3 days in regular medium.

### 2.3. Isolation and Identification of MSCs

Cut the umbilical cord of fresh and healthy full-term newborns into small sections of about 2 cm, rinsed them repeatedly in phosphate buffered saline (PBS) at 4°C until the blood stains were clean, visually inspected and the outer membrane was removed, separated the arteries and veins, and cut Wharton's jelly (WJ) tissue into small pieces of about 1 mm^2^. Spread the chopped umbilical cord tissue in a 25 cm^2^ culture flask with a density of about 50~ 60%. Three (3) ml of DMEM/F12 medium containing 10% FBS was added, cultured in an incubator at 37°C and 5% CO_2_ concentration, and the medium was changed every 3 days. When the cell density reached about 90%, they were washed twice with sterile PBS, digested with trypsin, centrifuged, and passaged. The MSCs used for experimentation were those obtained after the 3^rd^ to the 5^th^ passage. This study was approved by the ethics committee of the hospital (Y (2020) 010), and all the participants had signed informed consent forms.

The cell phenotypes of MSCs were analyzed by flow cytometry, washed the MSCs with PBS, and distributed to their respective flow cytometry tubes. Ten (10) *μ*l of flow cytometry antibody: HLA-DR-FITC, CD14-APC, CD19-FITC, CD34-APC, CD45-PerCP, CD73-PE, CD90-PE and CD105-PE. The cells were incubated at room temperature for 30 minutes. Subsequently, they were centrifuged at 1000 ×g 5 minutes to remove the supernatant. Three hundred (300) *μ*l PBS was used to resuspend the cells and detected with the FACS Canto flow cytometer (BD Biosciences, San Jose, CA, USA). The morphological characteristics of MSCs were observed through the optical microscope. The osteogenic as well as the adipogenic differentiation capabilities of MSCs was detected by Oil Red O staining and Alizarin Red staining, respectively, and the images were observed via microscopically.

### 2.4. Preparation of Conditioned Medium

When the fusion rate of MSCs reached 80%, the culture medium was discarded, and the cells were cultured in serum-free DMEM/F12 for another 48 hours. Then, the culture medium was collected, and the supernatant was collected by centrifugation at 1700 ×g 10 minutes at 4°C. The supernatant was filtered through a sterile filter (0.22 *μ*m) to obtain the MSC conditioned medium (CM) and stored at -80°C for subsequent experiments [16].

### 2.5. Isolation and Analysis of Exosomes

MSCs cultures using to isolate exosomes were grown in serum-free DMEM/F12 for 48 hours. Exosomes were isolated from MSC-CM collected by serial centrifugation as previously described [17, 18], and exosomes were resuspended in PBS. Exosomes were quantified by BCA protein quantification. The exosomal protein markers (CD9, CD63, CD81, and Alix) in MSCs and MSC-derived exosomes were detected by 10% sodium dodecyl sulfate–polyacrylamide gel electrophoresis (SDS-PAGE). Transmission electron microscopy was utilized to observe and analyze the morphology of the exosomes by negative staining. The size of exosome was identified by nanoparticle tracking analysis with the Zetasizer Nano ZS (Malvern Instruments, Malvern, UK).

### 2.6. Establishment of the Coculture System

**Figure 1:**
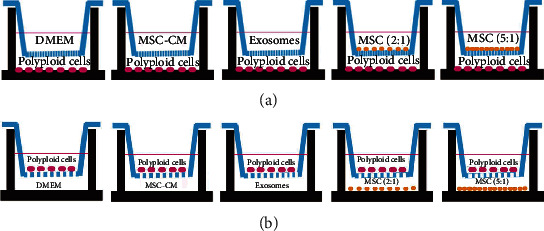
Schematic diagram of transwell coculture system and transwell migration assay. (a) Schematic diagram of the 0.4 *μ*m pore insert of the transwell coculture system. (b) Schematic diagram of the 8.0 *μ*m pore insert of the transwell migration assay.

The polyploid A549 and H1299 cell suspension, induced by Doc (3 × 10^4^/well), were each inoculated into different wells of two 6-well plate in a final volume of 2.5 ml complete DMEM/F12. After 16-18 hours, the cells adhered to the wall, they were replaced by MSC-CM containing 10% fetal bovine serum (as CM group) or MSC-exosomes containing 10% exosomes-free fetal bovine serum (as EXO group) and cultured for 48 hours. The polyploid A549 and H1299 cells were cocultured with MSCs in two-chamber dishes (Transwell system, Corning, NY, USA). The polyploid A549 and H1299 cells (3 × 10^4^/well) were seeded at the bottom of the transwell in 2.5 ml of complete DMEM/F12 media. After spreading the cells, the top insert (0.4 *μ*m pore size) of the transwell was seeded with 1.5 ml volume of MSCs (6 × 10^4^ cells/well as MSC 2 : 1 group or 1.5 × 10^5^/well as MSC 5 : 1 group) and cultured at 37°C with 5% CO_2_. The cells were cocultured for 48 hours, and the polyploid A549 and H1299 cells were obtained. Cells cannot penetrate the transwell insert and only culture medium, and signaling proteins are allowed to pass through. The polyploid A549 and H1299 cells were cultured in the complete DMEM/F12 as the control group (Figure 1(a)).

### 2.7. DNA Content Assay

The polyploid A549 and H1299 cells were harvested, suspended and washed in PBS, centrifuged (1000 g × 5 minutes), and fixed in 80% iced methanol at -20°C overnight. Then, cells were resuspended in 500 *μ*l PBS containing PI (50 *μ*g/ml) in the dark for 30 minutes at room temperature. DNA content assay were carried out by using the FACS Canto flow cytometer and was analyzed by BD FACSDiva Software.

### 2.8. Immunofluorescence Staining

The polyploid A549 and H1299 cells were washed with PBS and fixed with 4% paraformaldehyde. Then, the cells were gently washed three times with PBS and incubated for 1 hour in 5% bovine serum albumin (BSA) and 0.3% triton X-100. Cells were incubated with anti-LC3A/B antibody overnight at 4°C. Next, the cells were rinsed thrice with PBS and incubated with goat anti-mouse IgG and goat anti-rabbit secondary antibodies for 2 hours. After washing cells with PBS twice, the cells were counterstained with 2-(4-Amidinophenyl)-6-indolecarbamidine dihydrochloride (DAPI) for nucleus visualizing and photographed using a fluorescent microscope (Nikon, Japan).

### 2.9. Proliferation Assay

The polyploid A549 and H1299 cells were stained with 100 *μ*l of 2.5 *μ*M CFSE for 15 minutes at 37°C in the dark and washed twice with PBS. Afterwards, stained cells were cultured in a 6-well plate and grown at 37°C and 5% CO_2_ for 3 days. Cells were resuspended in PBS for flow cytometry-based analysis to measure CFSE staining at a wavelength of 488 nm. A reduction in fluorescence intensity can be correlated with increases in the rate of cell proliferation.

### 2.10. Transwell Migration Assay

The polyploid A549 and H1299 cells were plated in the upper chamber (Transwell system, Corning, NY, USA, 8 *μ*m pore size) at a density of 1 × 10^5^ cells/ml in complete medium for 24 hours to ensure cell adhesion. The wells in the lower chamber were filled with MSC-CM, MSC, or MSC-exosomes. After incubation for 48 hours, the upper chambers were fixed with 4% paraformaldehyde for 30 minutes and stained with crystal violet solution to each well for 10 minutes. The cells were washed with PBS and observed under an inverted microscope after drying. Five visual fields in the center and around were taken and counted (Figure 1(b)).

### 2.11. Real-Time Quantitative Polymerase Chain Reaction (RT-qPCR)

Total RNA was isolated by TRIzol Reagent from the polyploid A549 and H1299 cells. After detecting the concentration and purity of RNA by measuring the absorbance at 260 nm, real-time quantitative polymerase chain reaction (RT-qPCR) was performed on a RT-qPCR system using SYBR-Green I (ABI 7500, Takara). The expression of mRNA was normalized to the GAPDH mRNA expression. The primers sequences were recorded in Table 1.

### 2.12. Analysis of Apoptosis

Cell apoptosis was conducted by the Annexin-V-FITC/PI apoptosis kit. The polyploid A549 and H1299 cells were collected by trypsinization and incubated with 5 *μ*l Annexin V-FITC and 5 *μ*l PI for 15 minutes at room temperature. Stained cells were subjected to flow cytometric analysis using a FACSCalibur instrument (BD Biosciences) within 1 hour, and a total of 10 000 events were acquired and analyzed using the CellQuest software: the percentage of apoptotic cells = the percentage of early apoptotic cells (Q2) + the percentage of late apoptotic cells (Q4).

### 2.13. Western Blot

The polyploid A549 and H1299 cells were harvested and washed twice with precold PBS. The proteins from the cell lysate were extracted by radioimmunoprecipitation assay (RIPA) lysis buffer containing the protease and phosphatase inhibitor cocktails and ultrasonic disruption. The concentration of proteins was measured by the Pierce BCA Protein Assay Kit. Equal amount of protein sample (about 30 *μ*g) was subjected to 6-12% SDS-PAGE and then electrotransferred onto polyvinylidene membranes (Merck Millipore, USA). The membranes were blocked with TBS-T (50 mM Tris, 150 mM NaCl, 0.05% Tween-20) containing 5% nonfat milk for 2 hours at room temperature. This blocking step was followed by incubation in appropriate primary antibodies overnight at 4°C. After washing with TBS-T, the membranes were incubated with the indicated HRP-conjugated secondary antibodies for 2 hours at room temperature. After washing with TBS-T, SuperSignal West Femto Substrate was used to detect the protein expression. The images were captured on a photographic film. GAPDH was used as an internal control.

### 2.14. Statistical Analysis

All the statistical comparisons were performed using the GraphPad Prism 7.0 software. Statistical comparisons between multiple groups were performed using one-way analysis of variance (ANOVA) with Newman-Keuls multiple comparisons method. Statistical significance was defined as *p* < 0.05, and *p* values were indicated with asterisks in the figures as follows: ^∗^*p* < 0.05, ^∗∗^*p* < 0.01, and ^∗∗∗^*p* < 0.001.

## 3. Results

### 3.1. Doc Induced Formation of the Polyploid NSCLC Cells

**Figure 2:**
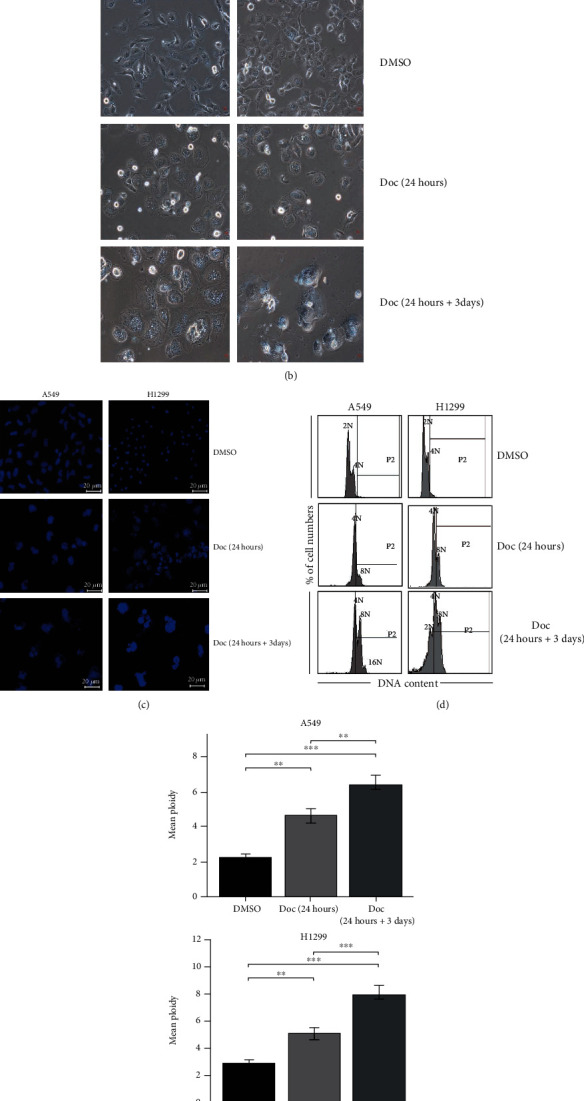
Doc induced formation of the polyploid NSCLC cells. (a) Experimental design. The NSCLC cell lines A549 and H1299 were treated with 1/0.5 *μ*M Doc for 24 hours to induce mitotic failure and then cultured in a drug-free medium for 3 days. Human umbilical cord-derived MCSs, MSC-CM, MSC-exosomes, and the polyploid A549 or H1299 cells were cultured together for 48 hours or 72 hours in indirect co-culture using the transwell system. (b) Morphologic change in A549 and H1299 cells by conventional light microscopy after treatment with1/0.5 *μ*M Doc for 24 hours and then cultured in a drug-free medium for 3 days (×200). (c) The morphology of the polyploid A549 and H1299 cells were observed by fluorescence microscopy; blue represented DAPI-labeled nucleus (×200). (d, e) DNA content of the polyploid A549 and H1299 cells was analyzed by flow cytometry.

The experimental design is shown in Figure 2(a). The NSCLC cell lines A549 and H1299 were treated with 1/0.5 *μ*M Doc for 24 hours to induce mitotic failure and then cultured in a drug-free medium for 3 days. The surviving A549 and H1299 cells entered an endoreplication cell cycle and grew as polyploid cells. The polyploid A549 and H1299 cells could be observed after removal of the floating dead cells in the Doc (24 hours+3 days) group, in contrast to the control cells (Figure 2(b)). Immunofluorescence results also showed that the cell volume of the Doc (24 hours+3 days) group was significantly increased, and the nucleus was multinucleated (Figure 2(c)). Flow cytometry analysis showed that Doc induced the polyploidy of A549 and H1299 cells, and the mean ploidy was (6.37 ± 0.49) N and (8.01 ± 0.50) N, significantly more than that in the DMSO group and Doc (24 hours) group (Figures 2(d) and 2(e), *p* < 0.05). All the following experiments were performed with Doc (24 hours+3 days) group cells as research objects unless stated otherwise.

### 3.2. Morphology and Phenotype Identification of MSCs and MSC-Exosomes

**Figure 3:**
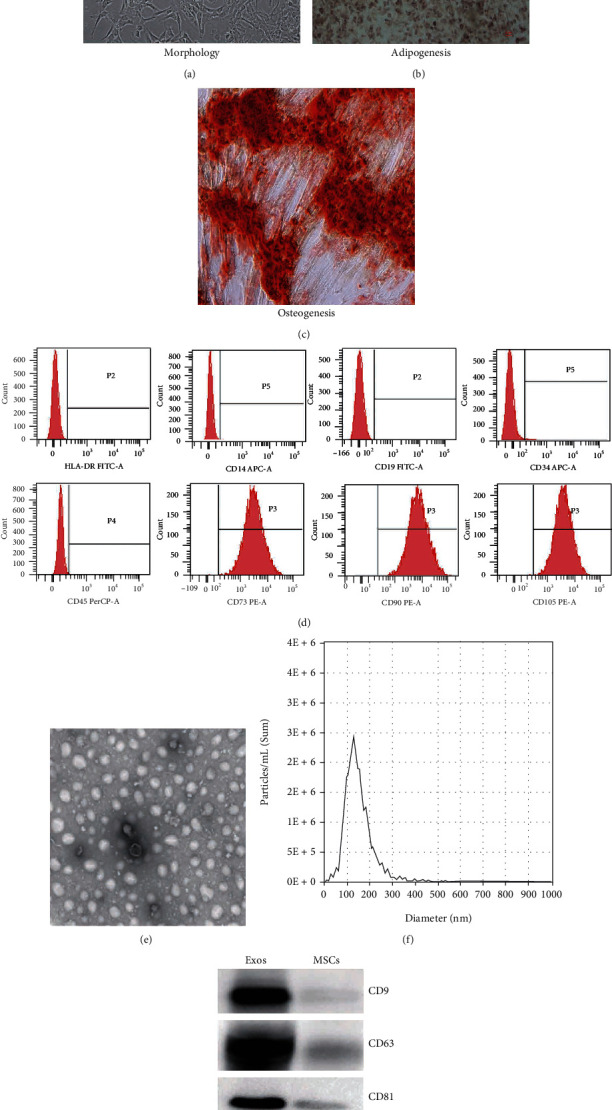
Characterization of umbilical cord-derived MSCs and MSC-exosomes. (a) Cell morphology of MSCs in single culture and MSCs was showing normal morphology in culture conditions (×100). (b, c) The differentiation potential of MSCs into two predominant lineages: adipogenesis and osteogenesis (×100). (d) The expression of surface markers of MSCs (HLA-DR, CD14, CD19, CD34, CD45, CD73, CD90, and CD105) was detected by flow cytometry. (e) The morphology of MSC-exosomes was observed under transmission electron microscopy (scale bar = 200 nm). (f) Particle size distribution of MSC-exosomes was analyzed by nanoparticle tracking analysis. (g) The expression of markers of exosome MSCs (CD9, CD63, CD81, and Alix) was detected by western blot.

Under the inverted microscope, MSCs were long and fusiform, with uniform size, compact cell clusters, swirling or parallel growth (Figure 3(a)). In addition, we found that the MSCs showed adipogenic and osteogenic differentiation, as demonstrated by Oil Red O staining (Figure 3(b)) and Alizarin Red staining (Figure 3(c)). Flow cytometry analysis of MSCs phenotype identification showed that the cells were negative for HLA-DR, CD14, CD19, CD34 and CD45 (negative rate< 5%), but were highly positive for CD73, CD90 and CD105 (positive rate> 95%) (Figure 3(d)), in line with the 2006 International Society for Cell Therapy (ISCT) standards for the identification of MSCs [19].

Transmission electron microscopy revealed MSC-exosomes as typically rounded nanoparticles (Figure 3(e)). Nanoparticle tracking analysis (NTA) exhibited a similar size distribution ranging from 100 to 200 nm in size (Figure 3(f)). Western blot revealed the presence of exosome surface markers, including CD9, CD63, CD81, and Alix (Figure 3(g)).

### 3.3. MSCs and MSC-Exosomes Promoted Migration and Epithelial-Mesenchymal Transformation (EMT) in the Polyploid A549 and H1299 Cells

**Figure 4:**
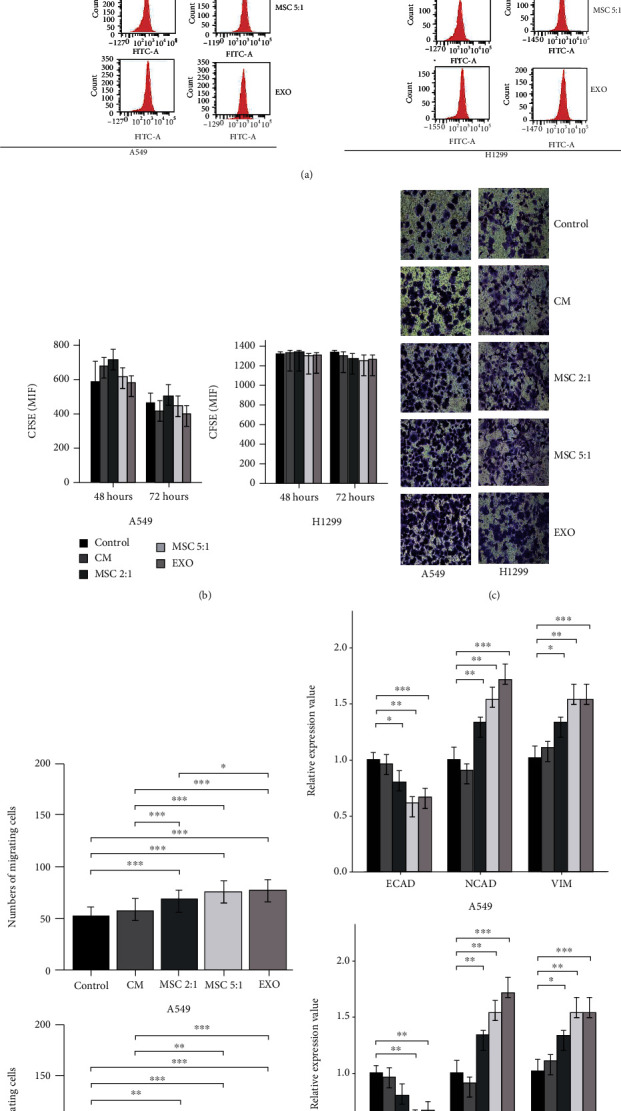
MSCs and MSC-exosomes promote migration and epithelial-mesenchymal transformation in the polyploid A549 and H1299 cells. (a, b) The CFSE proliferation analysis of the polyploid A549 and H1299 cells following coculture with MSC-CM, MSCs, and MSC-exosomes was examined by flow cytometry at 0 hour, 48 hours, and 72 hours. (c, d) The quantitative analysis of migration ability of the polyploid A549 and H1299 cells following coculture with MSC-CM, MSCs, and MSC-exosomes was examined by transwell assay at 48 hours. (e) The expressions of ECAD, NCAD, and VIM in the polyploid A549 and H1299 cells following c-culture with MSC-CM, MSCs, and MSC-exosomes were detected by RT-qPCR analysis at 48 hours.

We first evaluated the effects of MSCs and MSC-exosomes on the ability of polyploid A549 and H1299 cells to proliferate, migrate, and develop tumor characteristics. The proliferation of the polyploid A549 and H1299 cells was detected using CFSE assay. As shown in Figures 4(a) and 4(b), MSC-CM, MSCs, and MSC-exosomes had no effect on cell proliferation in the polyploid A549 and H1299 cells compared to the control group at 48 hours and 72 hours (*p* > 0.05). Transwell assay was conducted to investigate the impact of MSC-CM, MSCs, and MSC-exosomes on the cell migration ability of the polyploid A549 and H1299 cells. As shown in Figures 4(c) and 4(d), administering MSCs and MSC-exosomes significantly promoted the polyploid A549 and H1299 cell migration compared to the control group (*p* < 0.05). In the presence of MSCs and MSC-exosomes, the expression levels of mesenchymal gene markers including NCAD and VIM were increased, while the expression of the epithelial gene marker ECAD was reduced as shown by RT-qPCR analysis (*p* < 0.05). However, compared with the control group, the polyploid A549 and H1299 cells cocultured with the MSC-CM had no significantly change of migration and EMT (Figures 4(c)–4(e), *p* > 0.05). Taken together, these results demonstrated that MSCs and MSC-exosomes could enhance the migration and EMT of the polyploid A549 and H1299 cells.

### 3.4. MSCs and MSC-Exosomes Reduced Apoptosis and Promoted Autophagy in the Polyploid A549 and H1299 Cells

**Figure 5:**
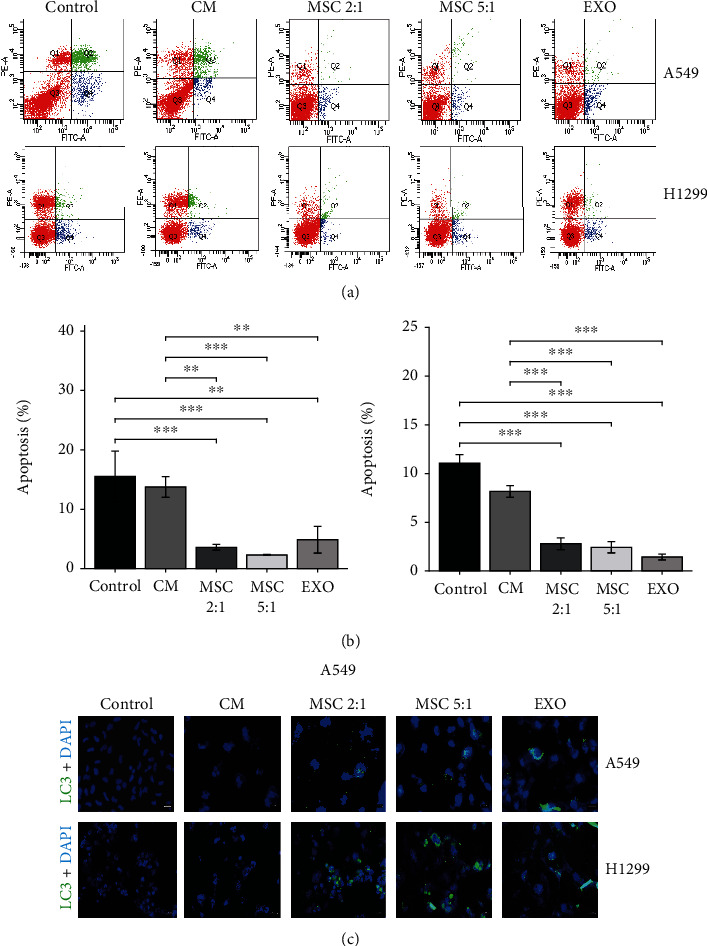
MSCs and MSC-exosomes reduced apoptosis and promoted autophagy in the polyploid A549 and H1299 cells. (a, b) Apoptosis analysis of the polyploid A549 and H1299 cells following coculture with MSC-CM, MSCs, and MSC-exosomes was examined by flow cytometry at 48 hours. (c) Stained with anti-LC3A/B antibody to detect the LC3 puncta and DAPI to detect nuclei by immunofluorescence using a fluorescence microscope (×200).

The capacity of cancer cells to escape apoptosis is one of the elements that make cancer a threatening disease. Hence, we investigated the ability of MSC-CM, MSCs, and MSC-exosomes to hinder the polyploid A549 and H1299 cells from undergoing apoptosis. The flow cytometry analysis profile was shown in Figures 5(a) and 5(b). There was a significant reduction in apoptotic cells of the polyploid A549 and H1299 cells, when cocultured with MSCs and MSC-exosomes, as compared to the polyploid A549 and H1299 cells cultured alone (Figures 5(a) and 5(b), *p* < 0.05).

Many researches have showed that autophagy had a double-edged effect on the development of NSCLC [20, 21]. Therefore, we determined the level of autophagy when MSC-CM, MSCs, and MSC-exosomes were cocultured within the polyploid A549 and H1299 cells by immunofluorescence and observed the formation of autophagy related protein LC3 under confocal microscopy. In Figure 5(c), compared with the control group, MSCs and MSC-exosome treatment augmented the LC3 puncta distribution in the polyploid A549 and H1299 cells but MSC-CM had no significantly change of the LC3 expression. The results suggested that MSCs and MSC-exosomes could promote autophagy in the polyploid A549 and H1299 cells.

### 3.5. MSCs and MSC-Exosomes Activated AMPK Signaling Pathway in the Polyploid A549 and H1299 Cells

**Figure 6:**
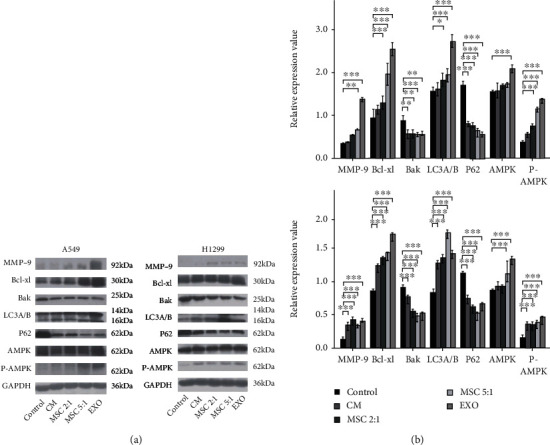
MSCs and MSC-exosomes activated AMPK signaling pathway in the polyploid A549 and H1299 cells. (a, b) Protein levels of MMP-9, Bcl-xl, Bak, LC3A/B, P62, AMPK, and phospho-AMPK (Thr172) in the polyploid A549 and H1299 cells following coculture with MSC-CM, MSCs, and MSC-exosomes were determined by Western blot analysis.

Autophagy is regulated by multiple pathways, and it was confirmed that the activation of AMPK signaling pathway could also induce autophagy [22]. Compared with the control group, the expression of p-AMPK was increased in the polyploid A549 and H1299 cells treated by MSC-CM, MSCs, and MSC-exosomes (Figures 6(a) and 6(b)). Furthermore, the expressions of autophagy proteins such as LC3A/B was increased, and P62 was attenuated in the polyploid A549 and H1299 cells, when cocultured with MSC-CM, MSCs, and MSC-exosomes. The expression of metastasis associated protein MMP-9 was increased. In addition, the expression of survival proteins Bcl-xl was increased, and apoptosis proteins Bak was reduced (Figures 6(a) and 6(b)). These results suggested that MSCs could promote autophagy, migration, and induced antiapoptotic effect in the polyploid A549 and H1299 cells by the possible activation of the AMPK signaling pathway.

## 4. Discussion

There are quite a number of side effects after the treatment of NSCLC via important methods such as chemotherapy and radiotherapy [23]. Simultaneously, they also significantly increase the proportion of the polyploid cancer cells in patients [24]. Chelidonis et al. found that 71.1% (32/45) of the tumors of NSCLC patients were classified as aneuploidy. The DNA ploidy correlates with poor and moderately differentiated tumors [25]. Maounis et al. reported that DNA ploidy provided an independent prognostic parameter for patients with NSCLC [26]. The polyploid cancer cells have been regarded as senescent cells, but recent studies have shown that these polyploid cancer cells were actually alive and could produce daughter cancer cells [27, 28]. Mittal et al. reported that most of the prostate cancer (PC-3) cells exposed to Doc undergo cell death following mitotic arrest. However, a small number of cells that “slipped” from mitosis and survived for several weeks, formed huge multinucleated polyploid (MP) cells. These cells were resistant to chemicals (such as Doc) and eventually underwent asymmetric cell division or neogenesis to form small mononuclear aneuploid cells. Animal experiments showed that these MP cells had tumorigenic potential in nude mice. Therefore, MP cells, once considered to be in ultimate growth arrest or cell death, might represent a “transitional state” and produce viable progeny cells. Consequently, these cells may play a critical role in tumor recurrence and metastasis [29].

In recent years, MSCs and MSC-exosomes have become the focus of tumor biotherapy research. Recently, many researchers have shown great interest in the application of MSCs and MSC-exosomes in the treatment of the side effects caused by radiotherapy and chemotherapy in cancer patients. Zheng et al. found that the rat bone marrow MSCs could effectively reduce inflammation and fibrosis in the wounded skin of rats and promote the repair of acute radioactive skin injury [30]. Lei et al. reported that MSC-derived extracellular vesicles attenuated radiation-induced lung injury via miRNA-214-3p in mice [31]. However, the tumor-suppressing or tumor-promoting effects of MSCs have been the subject of controversy in the past decade. Studies have shown that transplanted MSCs can promote the growth and angiogenesis of tumor cells and inhibit the proliferation of T cells, the proliferation and killing activity of NK cells, and the secretion of cytokines, thereby playing a role in promoting tumor growth [32–34], but MSCs have inhibitory effects on various tumor models such as pancreatic cancer, liver cancer, prostate cancer, and colon cancer [35–38]. There are similar controversies about the role of MSC-CM and MSC-exosomes. Bone marrow MSC-CM has been shown to have antitumor effects on NSCLC cells but has no effect on glioblastoma cancer subsets [39]. These findings confirm that MSCs could promote tumor growth in some types of cancer, but inhibit invasion and metastasis in other cancers.

At present, the effect of MSCs on tumor biological behavior is mainly focused on the effect of MSCs on common cancer cells [40, 41]. Luo et al. showed that MSCs promoted autophagy activation, reactive oxygen species production, and EMT as well as increased migration and invasion in A549 cells [42]. No studies have been conducted on the direct effects of MSC on polyploid cancer cells. In this study, we analyzed the effects of MSCs and their derived exosomes on the biological behavior of the polyploid NSCLC cells. The results showed that although MSC-CM, MSCs, and MSC-exosomes had no effect on the proliferation of the polyploid A549 and H1299 cells, MSCs and MSC-exosomes could significantly promote the migration, antiapoptosis, and autophagy of the polyploid A549 and H1299 cells. EMT is a process in which epithelial cells lose polarity and transform into mesenchymal cells [43]. This process reduces cell-cell adhesion and increased cellular mobility, invasiveness, stem cell-like properties, and antiapoptotic ability [44]. Increasing evidence supports the existence of intermediate EMT states characterized by the concomitant presence of both epithelial and mesenchymal features, offering a more dynamic interpretation of the fluidity and plasticity of the EMT program. Indeed, intermediate EMT states have been identified as key drivers of cancer progression [45, 46]. Our data showed that MSCs and MSC-exosomes could increase the expression of mesenchymal markers NCAD and VIM but decrease the expression of epithelial marker ECAD. These results suggest that MSCs and MSC-exosomes promote EMT in the polyploid NSCLC cells. Although some literatures have reported the preparation methods of MSC-CM [47, 48], there is no gold standard method for the preparation of MSC-CM. If the protocol (especially the preparation of MSC-CM) changes, it may change the profile of the molecules released/paracrine factors, which may alter the final biological behavior of the polyploid cancer cells.

**Figure 7:**
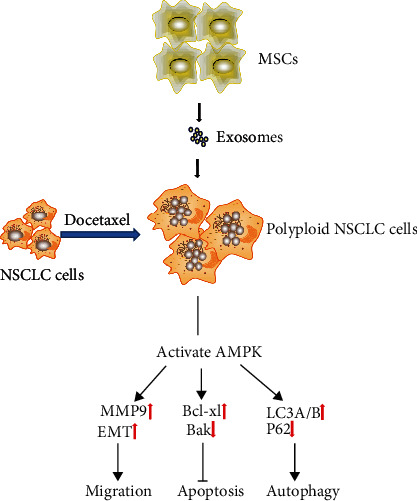
MSCs and MSC-exosomes may promote migration, antiapoptosis, and autophagy of polyploid NSCLC cells through the activated AMPK signaling pathway.

To clarify the possible molecular mechanisms of the polyploid NSCLC cells in response to MSCs and MSC-exosomes treatment, we determined the expression levels of migration, apoptosis, and autophagy-related proteins by Western blotting. AMPK is a highly conserved regulator of cell energy metabolism [49]. Activation of AMPK can reduce ATP utilization and activate autophagy [50]. Our study showed that MSCs might promote autophagy, migration, and antiapoptosis of the polyploid A549 and H1299 cells by activating the AMPK signaling pathway (Figure 7). However, the role of MSCs in polyploid NSCLC cells is still in its infancy. The specific mechanism of MSC effect on polyploid NSCLC cells remains to be further studied. Further, the application of MSCs and their derived exosomes in tissue and organ damage induced by radiotherapy and chemotherapy of NSCLC remains to be further evaluated.

## 5. Conclusion

In conclusion, our studies showed that MSCs and MSC-exosomes enhanced migration, antiapoptosis, and autophagy of the polyploid NSCLC cells through the AMPK signaling pathway, but our results are constrained by the experimental design and the model developed and characterized. Other cells in the tumor microenvironment (e.g., cancer-related fibroblasts and cancer stem cells) may affect the biological behavior of the polyploid cancer cells in the lung by affecting MSCs.

## Figures and Tables

**Table 1:** mRNA primer information.

Gene name	Forward primer (5′-3′)	Reverse primer (5′-3′)
ECAD	GCCTCCTGAAAAGAGAGTGGAAG	TGGCAGTGTCTCTCCAAATCCG
NCAD	CCTCCAGAGTTTACTGCCATGAC	GTAGGATCTCCGCCACTGATTC
VIM	AGGCAAAGCAGGAGTCCACTGA	ATCTGGCGTTCCAGGGACTCAT
GAPDH	GGTGAAGGTCGGAGTCAACG	CAAAGTTGTCATGGATGHACC

## Data Availability

The data used to support the findings of this study are available from the author upon request.
